# Clinical observation of individualized nutritional formula on inflammation index, immune status and gastrointestinal tolerance in patients with severe head injury

**DOI:** 10.12669/pjms.37.4.3987

**Published:** 2021

**Authors:** Chunying Zhu, Yingfu Zhang, Wei Li, Qianqian Li

**Affiliations:** 1 Chunying Zhu, Department of Neuroscience Critical Care Unit, Baoding No.1 Central Hospital, Baoding, 071000, Hebei, China; 2 Yingfu Zhang, Endoscopic Diagnosis and Treatment Center, Baoding No.1 Central Hospital, Baoding, 071000, Hebei, China; 3 Wei Li, Department of Neuroscience Critical Care Unit, Baoding No.1 Central Hospital, Baoding, 071000, Hebei, China; 4 Qianqian Li, Department of Neuroscience Critical Care Unit, Baoding No.1 Central Hospital, Baoding, 071000, Hebei, China

**Keywords:** individualized nutritional formula, severe head injury, inflammatory factors, immune status, gastrointestinal tolerance

## Abstract

**Objectives:**

To evaluate the clinical significance of individualized nutritional formulas on inflammatory factors, immune status and gastrointestinal tolerance in patients with severe head injury.

**Methods:**

A total of 80 patients with severe head injury who were hospitalized in Baoding No.1 Central Hospital from March 2017 to March 2020 were randomly divided into two groups with 40 cases in each group. Patients in both groups were given enteral nutrition (EN), the control group was given conventional enteral nutrition formula through nasointestinal tube, and the experimental group was given individualized nutrition formula. All patients were tested for tumor necrosis factor(TNF-α), C-reactive protein(CRP), interleukin 6(IL-6), IgA, IgM, IgG, serum intestinal fatty acid binding protein(I-FABP) and D-lactic acid concentration before and after enteral nutrition treatment. The incidence of adverse reactions such as abdominal distension, diarrhea, constipation, and gastric retention within seven days after treatment of two groups were compared and analyzed.

**Results:**

There was no significant difference in inflammatory factors such as TNF-a, CRP, IL-6, immunoglobulin levels, I-FABP and D-lactic acid concentration between the two groups before treatment (p>0.05). After treatment, the above indicators of the two groups of patients were better than before treatment, the difference was statistically significant (p<0.05), and the experimental group was significantly better than the control group (p<0.05). The experimental group had a gastrointestinal adverse reaction rate of 10%, and the control group had 27.5%, the difference was statistically significant (p=0.04).

**Conclusions:**

Individualized nutritional formula has more significant advantages than conventional nutritional formula for patients with severe head injury, which can reduce inflammatory response, increase the patient’s immune level, improve the intestinal mucosal barrier function, have good gastrointestinal tolerance, and have a low incidence of adverse reactions.

## INTRODUCTION

Severe head injury refers to severe damage to brain tissue caused by external forces. It is a common traumatic disease in the Department of Critical Care Medicine, with a high mortality and disability rate.^1^ Patients with severe head injury mostly have disturbances in consciousness, and their voluntary energy intake disappears.^2^ Patients with severe head injury are often accompanied by stress ulcers and gastrointestinal mucosal hypoperfusion, and they may cause gastrointestinal mucosal barrier dysfunction and limited nutrient absorption, which is extremely detrimental to their nutrition and rehabilitation. In addition, the inflammatory response after injury may lead to the release of great inflammatory factors, leading to further aggravation of brain tissue damage.^3^ Adequate enteral nutrition support at an early stage has positive significance for preventing hypoproteinemia, reducing lung infection, reducing patient stress response, adjusting metabolic mechanism, and improving prognosis.^4^ However, consensus on the formulation of enteral nutrients for patients with a severe head injury is unavailable, and the optimal content of nutrients and total nutritional requirements are still unclear. Currently, there is no effective and best method for monitoring.^5^ It is undoubtedly very meaningful to formulate individualized nutritional formulas and make certain adjustments for different patients.

In this study, we used individualized nutrition formulas for enteral nutrition for patients with severe head injury. From the perspective of inflammation indicators, immune status, and gastrointestinal tolerance, the individualized nutritional formula and conventional enteral nutrition were compared and analyzed to prove that the former has considerable advantages and is worthy of clinical promotion. Now report the specific situation as follows.

## METHODS

### Ethical Approval

The study was approved by the Institutional Ethics Committee of Baoding No.1 Central Hospital, and written informed consent was obtained from all participants.

### Inclusion criteria:

1. Clinically diagnosed as craniocerebral injury by CT or MRI.^6^
2. Aged less than 70 years old.
3. Had severe head injury (GCS score of 3-8).^7^
4. Had clear signs of positive nervous system.
5. Had family members agreed to the research plan and signed the consent form and were able to cooperate with the research work.

### Exclusion Criteria:

1. Had unstable hemodynamics.
2. Combined with severe gastrointestinal injury or bleeding who were not suitable for enteral nutrition.
3. Combined with severe dysfunction of important organs and could not be improved after active correction.
4. Combined with tumors or autoimmune diseases.
5. Be intolerant to enteral nutrition.
6. Had drugs affecting the research such as hormones and immunosuppressants in the near future.

A completely random design was adopted in this study. And the sample size of this study was obtained by querying the sample size table. A total of 80 patients with severe head injury admitted to Baoding No.1 Central Hospital from March 2017 to March 2020 were randomly divided into two groups according to the random number table method, with 40 patients in each group. The experimental group included 25 males and 15 females, aged 17-65 years old, with an average of 45.55±15.33 years old, and the control group included 23 males and 17 females, aged 23-67 years old, with an average of 46.45±13.96 years old. There was no significant difference in the general data of the two groups of patients, and the groups were comparable (Table-I).

**Table-I:** Comparative analysis of general data of experimental group and control group (*X̅* ±S) n=40.

Index	Experimental group	Control group	t/χ^2^	P
Age (years)	45.55±15.33	46.45±13.96	0.27	0.78
Male (case %)	25(62.5%)	23(57.5%)	0.21	0.64
BMI (kg/m^2^)	26.13±3.07	25.47±2.61	1.04	0.30
GCS score	6.46±1.21	6.75±2.07	0.76	0.45
APACHE II score	21.12±2.11	20.25±3.12	1.46	0.15

P>0.05

### Enteral Nutrition Therapy

The general treatment plan for both groups of patients included close monitoring and control of intracranial pressure, sedation of pain, supportive therapy, prevention of infection, stable cerebral perfusion, etc.^8^ The control group was given enteral nutrition (EN) through nasointestinal tube based on programs described in *ASPEN (American Society for Parenteral and Enteral Nutrition)*^9^, as below: First, keep the hemodynamic stable; apply an infusion pump to continuously pump enteral nutrition at an initial rate of 25ml/h; within three days the calorie volume reaches 25-30kcal/(kg.d), protein reaches 1.5-2.0g/(kg.d), and the blood glucose is controlled at 7.8 ~11.1mmol/L; dynamically evaluate the tolerance; evaluate the gastric residual every six hours; in case of <200mL, increase the EN pumping rate; in case of 200ml-500ml, maintain the original pumping rate; in case of the gastric residual≥500ml or accompanied by severe diarrhea, vomiting or aspiration, then slow down or suspend EN pumping or replace EN preparations. If this is still not tolerated, change to parenteral nutrition and terminate the study on this patient.

The experimental group was given a more refined and individualized nutritional formula based on the EN program. For patients with digestive dysfunction, nutritional formulas rich in amino acids or short peptides were selected, and for patients with normal digestive tract function, ensure nutrition containing dietary fiber were selected. For diabetics, low-sugar and rich nutrients such as dietary fiber, monounsaturated fatty acids and fructose were selected, and short-chain fatty acids, soluble dietary fiber, and live bifidobacterium preparations were added to the formula to adjust the intestinal flora and improve the function of the gastrointestinal tract. For patients with urinary disorders, insoluble dietary fiber was added. For patients with anemia, iron, folic acid, vitamin B12, vitamin C and a variety of trace elements were added.

### Observation indicators

1) Peripheral venous blood was sampled in the basal state in the morning before intervention at admission and 1d, 7d, and 14d after enteral nutrition therapy. Enzyme-linked immunosorbent assay (ELISA) was used to detect the change of inflammatory factors such as tumor necrosis factor (TNF-a), C-reactive protein (CRP), and interleukin 6 (IL-6); 2) Immune status: Peripheral venous blood was sampled in the basal state in the morning before treatment and 14 days after treatment to detect the immunoglobulins IgA, IgM, and IgG, and analyze their changes. Meanwhile, serum intestinal fatty acid binding protein (I-FABP) and D-lactic acid concentration levels were detected to evaluate the intestinal mucosal barrier function; 3) Gastrointestinal tolerance: The incidence of adverse reactions such as abdominal distension, diarrhea, constipation, and gastric retention within seven days after treatment was compared between the two groups.

### Statistical Analysis

SPSS 20.0 software was used to calculate all the data, and the measurement data was expressed as (*X¯*±S). Independent-samples T test was used for analysis between groups, repeated measures analysis of variance was used for data analysis within groups, and χ^2^ test was used for comparison of rates. The significance level α was set to 0.05 and the confidence interval was 95%.

## RESULTS

Changes of inflammatory factors in both groups before and after treatment are shown in Table-II, indicating that TNF-a, CRP and IL-6 in both groups were significantly increased before treatment and the difference was not significant (p>0.05); after treatment, the indicators above were lower than those before treatment, and the difference was statistically significant (p<0.05). TNF-a and IL-6 in the experimental group and the control group showed no significant difference at day-one after treatment (TNF-a, p=0.52; IL-6, p=0.33); and seven days and 14d after treatment, the results in the experimental group were significantly lower than those in the control group, with the difference significant (p=0.00). CRP in the experimental group was significantly lower than that in the control group at 1d after treatment (p=0.00), and it decreased significantly at 7d and 14d (p=0.00).

**Table-II:** Comparative analysis of changes in inflammatory factors before and after treatment in both groups (*X̅*±S) n=40.

Group		Before treatment*	1d after treatment	7d after treatment Δ	14d after treatment Δ	F	P
TNF-ɑ(ng/L)	Experimental group Δ	46.32±12.27	27.35±11.43	6.77±1.04	4.02±2.21	21.25	0.00
	Control group Δ	45.53±11.57	28.54±11.51	11.53±4.35	7.15±3.04	20.29	0.00
	t	0.67	0.65	4.31	4.08		
	p	0.31	0.52*	0.00	0.00		
CRP(mg/L)	Experimental group Δ	44.72±7.41	16.76±5.03	6.47±1.51	4.31±0.77	34.31	0.00
	Control group Δ	44.53±7.06	23.05±6.34	10.21±5.33	5.22±1.22	34.78	0.02
	t	0.65	4.92	13.42	4.25		
	p	0.23	0.00	0.00	0.00		
IL-6(ng/L)	Experimental group Δ	15.31±5.25	10.23±1.72	7.21±2.07	2.76±0.13	15.11	0.00
	Control group Δ	17.33±4.68	10.55±1.18	9.33±2.53	5.25±1.42	15.62	0.00
	t	1.82	0.97	4.10	11.04		
	p	0.07	0.33*	0.00	0.00		

* p>0.05, Δp<0.05.

The immunoglobulin levels of both groups of patients improved after treatment compared with before treatment (p=0.00), and the experimental group improved more significantly than the control group after treatment, and the difference was statistically significant (IgG, IgA, p=0.01; IgM, p=0.00) (see Table-III).

**Table-III:** Comparative analysis of immunoglobulin levels before and after treatment of groups(*X̅*±S) n=40.

Observational index		IgG(g/L)				IgA(g/L)				IgM(g/L)		

Group	Before treatment *	After treatment Δ	t	p	Before treatment *	After treatment Δ	t	p	Before treatment *	After treatment Δ	t	p
Experimental group Δ	7.79±2.13	13.25±3.31	7.17	0.00	1.17±0.38	2.76±1.43	5.09	0.00	1.43±0.78	2.55±0.84	6.18	0.00
Control group Δ	8.73±4.25	11.13±4.06	2.58	0.01	1.22±0.46	2.15±0.48	8.85	0.00	1.36±0.24	2.12±0.23	14.46	0.00
t	1.25	2.56			0.53	2.56			0.54	3.12		
p	0.21	0.01			0.60	0.01			0.60	0.00		

* p>0.05, Δp<0.05.

The intestinal mucosal barrier function indexes of both groups were improved after treatment compared with before treatment (p=0.00), and the experimental group improved more significantly than the control group after treatment, and the difference was statistically significant (I-FABP, p=0.01; D-lactic acid, p=0.00) (Table-IV).

**Table-IV:** Comparative analysis of intestinal mucosal barrier function indexes before and after treatment of both groups (*X̅*S ) n=40.

Observational index	I-FABP (ug/L)				D- lactic acid(ug/L)			

Group	Before treatment *	After treatment Δ	t	p	Before treatment *	After treatment Δ	t	p
Experimental group Δ	71.29±9.13	19.25±3.36	34.16	0.00	100.27±10.31	32.36±3.47	39.48	0.00
Control group Δ	68.73±9.25	21.13±3.08	30.87	0.00	103.22±10.46	43.75±4.48	33.05	0.00
t	1.25	2.61			1.27	12.71		
p	0.21	0.01			0.20	0.00		

* p>0.05, Δp<0.05.

**Table-V:** Comparative analysis of intestinal tolerance after treatment of both groups (*X̅* ±S) n=40.

Group	Abdominal distension	Diarrhea	Constipation	Gastric retention	Total	Incidence
Experimental group	1	1	0	2	4	10%
Control group	2	4	3	2	11	27.5%
χ^2^						4.02
p						0.04

p<0.05.

Within seven days of treatment, the incidence of gastrointestinal adverse reactions in the experimental group was 10% and that in the control group was 27.5%. The gastrointestinal tolerance of the experimental group was significantly better than that of the control group, and the difference was statistically significant (p=0.04).

## DISCUSSION

Nutritional treatment of severe head injury was contradictory to a certain extent. On the one hand, the body was in a state of stress after the injury. The release of stress hormones such as catecholamines and glucagon made the body in a state of high metabolism, and energy consumption was abnormally increased.^10^ On the other hand, patients with severe head injury were often unable to take food on their own, leading to a lack of nutrients, and the abnormal glucose and lipid metabolism, hypoproteinemia, etc. might cause adverse consequences. Therefore, reasonable nutritional support after injury was of great significance for patients to reduce hypoproteinemia and hypermetabolic state, and to promote recovery.^11^

In clinical practices, common nutritional support includes parenteral and enteral nutrition. A meta-analysis suggested that early enteral nutrition support after head injury had obvious advantages in improving the prognosis. Our research results showed that feeding through small intestinal and the immune enhancement formula could reduce infectious complications.^12^ Compared with parenteral nutrition, enteral nutrition was more in line with the physiological state of the normal human body. Enteral nutrition could ensure the blood supply of intestinal wall through the stimulation of enteral nutrients, promote intestinal peristalsis, maintain the permeability and structural and functional integrity of intestinal mucosa, and avoid the movement of intestinal flora to maintain the barrier function of intestinal mucosa. The energy of brain tissue was mainly derived from glucose. Enteral nutrition could increase the concentration of blood sugar and increase the intake of brain tissue, which was of great significance for the repair of damaged brain tissue.^13^ Meanwhile, nutrients could be absorbed into the liver through the portal vein to promote protein synthesis and regulate high metabolic reactions.^14^ Reintam et al. believed that^15^ patients without severe complications such as uncorrectable shock, hypoxemia and acidosis, uncorrectable bleeding, intestinal ischemia or intestinal obstruction, should be given enteral nutrition.

The current enteral nutrition methods mainly included continuous enteral nutrition, intermittent enteral nutrition and compound nutrition support. However, Mazaherpur’s research showed that^16^ none of the three methods could meet the energy needs of patients. While the continuous method had a positive effect on nitrogen balance, high metabolism reduction and total protein maintenance in the body, and was the preferred method for patients with brain injury. There were many types of enteral nutrition, but consensus on enteral nutrition for patients with a severe head injury was still unavailable. The views of medical and nursing staff might be crucial for patients to receive nutritional therapy, but current research on this aspect was also very limited. During the entire treatment process, close observation of the patient by medical staff and adjustment of the nutritional plan at any time were very important for the recovery of the patient.^17^ A study from the European Centre for Neuronutrition showed^18^ that there were great differences in preferences for outcomes of nutritional support measures. Good practices were more beneficial to patients and had a significant impact on the outcome of treatment. Curtis et al.^19^ also suggested that a variety of nutritional strategies were essential for the recovery of brain injury.

Organs and tissues of patients with severe head injury were in a state of low function.^20^ In addition, inflammatory factors caused by injury were activated, leaving patients in a state of low immune function and high inflammation, which was very unfavorable for the prognosis of patients. Improving the immune function of patients and reducing the negative interference of inflammatory factors were beneficial to the prognosis of patients. Some patients would have improved nutritional indicators and infection rates when receiving immune-enhancing additives (such as glutamine, arginine, and omega-3 fatty acids).^21^ Lorenz et al.^22^ suggested that compared with patients receiving an isocaloric diet, patients receiving glutamine supplementation had better total lymphocyte count, activated CD4+DR+T helper lymphocyte percentage, lymphocyte response to mitogen in vitro, and normalization rate of IL-2 plasma levels. We believed that it was completely reasonable to provide a demand-oriented immune stimulation diet for critically ill patients, because it could reduce sepsis complications, speed up wound healing, and shorten the length of stay in the intensive care unit (ICU) and general ward. In a study involving 67 patients, the effects of probiotics combined with early enteral nutrition on the levels of endothelin-1 (ET-1), C-reactive protein (CRP), and inflammatory factors in patients with severe traumatic brain injury (TBI) were investigated, suggesting that probiotics combined with early enteral nutrition could reduce serum ET-1, CRP, IL-6, IL-10, and TNF-α levels in patients with severe TBI, thereby increasing the recovery rate of patients.^23^ Horn et al. believed that^24^ patients receiving high-protein formula enteral nutrition (more than 20% of calories) had a 25% higher recovery rate than those receiving normal protein content, suggesting that clinicians should strongly consider using a formula containing at least 20% of protein when possible, rather than the standard formula.

In this study, individualized nutritional formula was applied to treat patients with a severe head injury. In addition to conventional nutrients in the nutritional formula, the formulas of different patients were also adjusted according to the literature, such as adding dietary fiber, protein content, monounsaturated fatty acids, fructose, etc. At the same time, the beneficial live bacteria preparation and trace elements were added. Better clinical effects have been obtained. Compared with the traditional nutritional formula, both groups had significantly reduced inflammatory factors such as TNF-a, CRP, IL-6 after treatment, and the difference was significant (p=0.00). After treatment, the immunoglobulin level of the experimental group was significantly improved (IgG, IgA, p=0.01; IgM, p=0.00), and the intestinal mucosal barrier function was significantly improved (I-FABP, p=0.01; D- Lactic acid, p=0.00). And the individualized nutritional formula was adjusted according to the patient’s gastrointestinal condition, the incidence of gastrointestinal adverse reactions was lower and the tolerance was better (p=0.04).

### Limitations of this study

This study still has shortcomings. In this study, there are few cases and short follow-up time. No further examination and analysis are performed after the occurrence of gastrointestinal adverse reactions to clarify the causes of adverse reactions. We are constantly increasing the sample size and follow-up time in order to elaborate on the shortcomings and long-term effects of individualized nutritional formulas, so that more patients can benefit from it.

## CONCLUSIONS

Individualized nutritional formula has more significant advantages than conventional nutritional formula for patients with severe head injury, which can reduce inflammatory response, increase the patient’s immune level, improve the intestinal mucosal barrier function, have good gastrointestinal tolerance, and have a low incidence of adverse reactions. It is worthy of clinical application.

### Authors’ Contributions:

**CZ & YZ:** Designed this study and prepared this manuscript, and are responsible for the accuracy and completeness of the manuscript.

**WL:** Collected and analyzed clinical data.

**QL:** Significantly revised this manuscript.
